# Effectiveness of LiuZiJue Qigong versus traditional core stability training for poststroke patients complicated with abnormal trunk postural control: study protocol for a single-center randomized controlled trial

**DOI:** 10.1186/s13063-020-4117-6

**Published:** 2020-03-12

**Authors:** Chen Wang, Long Yu, Jian Yang, Ren Wei Wang, Ya Nan Zheng, Ying Zhang

**Affiliations:** 1 Department of Rehabilitation, Shanghai Xuhui Central Hospital, No. 966 Middle Huaihai Road, Xuhui District, Shanghai, 200031 People’s Republic of China; 2 School of Kinesiology, Shanghai University of Sport, No. 200 Hengren Road, Yangpu District, Shanghai, 200438 People’s Republic of China

**Keywords:** Six-character formula, Trunk postural control, Stroke, Core stability, Traditional core stability training, LiuZiJue Qigong (LQG)

## Abstract

**Background:**

Trunk function in stroke patients with hemiplegia is associated with respiration and core stability and is also found to be associated with balance and postural control and activities of daily living. LiuZiJue Qigong (LQG) is a traditional Chinese method of fitness based on breath pronunciation. The purpose of this study is to compare the clinical efficacy of LQG and traditional core stability training in the treatment of stroke patients with abnormal trunk posture. This protocol is written according to the SPIRIT 2013 statement.

**Methods/design:**

This study is a single-center randomized controlled trial in which 160 stroke patients are randomly divided into a study group and a control group. Patients in the study group will receive LQG combined with conventional rehabilitation therapy, and patients in the control group will receive traditional core stability training combined with conventional rehabilitation therapy. All treatments will be done for 45 min/day, five times per week, for 2 weeks. The primary outcome (Trunk Impairment Scale) and secondary outcomes (Berg Balance Scale, Fugl-Meyer Assessment, Modified Barthel Index, Maximum Phonation Time, Dynamic and Static balance testing, and thickness and the mobile degrees of diaphragm) will be measured at baseline, 2 weeks, and the end of the rehabilitation course.

**Discussion:**

The aim purpose of this research study is to compare the clinical efficacy of LQG and traditional core stability exercise in the treatment of stroke patients with abnormal trunk posture.

**Trial registration:**

Chinese Clinical Trial Register, ChiCTR1800014864. Registered on 24 November 2018.

## Background

Several studies have shown that trunk posture control disorder is common in patients with hemiplegia after stroke [1]. Therefore, the problem of trunk control disorder in stroke patients should receive more attention in the course of rehabilitation. It is characterized by trunk coordination and sitting balance problems, trunk posture control and trunk muscle strength, and trunk position perception [2]. “Postural control” refers to the ability to keep the body in appropriate space during all kinds of physical activities [3]. Correct postural control depends on valid core stability. The core muscle group is divided into the deep muscle and shallow movement muscle groups, in which the deep muscle group should play the main role in stabilizing the trunk [4]. The deep core muscles, such as the diaphragm, the abdominal transverse muscle, and the pelvic floor muscle, are also the main muscles responsible for breathing [5, 6]. For example, during inhaling, diaphragm centripetal contraction, diaphragmatic roof fall, abdominal and pelvic floor muscle centrifugal contraction, and increased intra-abdominal pressure occur, enhancing core stability. Obviously, deep core muscles are closely related to posture control and breathing ability [7, 8].

Currently, core stability training is often used as a clinical treatment for stroke. However, respiratory training is often neglected in the rehabilitation of stroke. To maintain the core stability of stroke patients with hemiplegia, the chest, pelvis, and abdominal cavity are in abnormal positions, and the breathing movements of the diaphragm are affected, resulting in overinvolvement of the auxiliary respiratory muscles, which exacerbates postural abnormalities. Therefore, it is very important to explore a treatment approach that combines respiration and trunk control to improve a stroke patient’s ability to control the trunk.

Inspiration is related to trunk extension, and exhalation is related to trunk flexion. Patients with hemiplegia often show abnormal chest uplift, collapsed abdominal muscle force on the hemiplegic side, and overextension of the trunk. Abnormal trunk posture control in poststroke patients could lead to abnormal breathing. Fugl-Meyer et al. found that decreased expiratory force is a common feature in stroke patients with hemiplegia [9]. In subsequent studies, it was also found that “abdominal electromyography decreased during forced exhalation” [10]. Patients with cerebral apoplexy generally have abnormal breathing, which is often manifested as decreased exhalation ability [11]. Because of all these factors, proper breathing exercises, especially exhalation exercises, are necessary in patients with stroke [12].

LiuZiJue Qigong (LQG) is a traditional fitness method that focuses on controlling breathing. It is part of a new fitness qigong series launched by the Chinese Health Qigong Association [13]. LQG involves performing the actions of inhaling and exhaling through different mouth patterns to regulate and control the rise and fall of the breath in the body, and completing the practice of “xu, he, hu, si, chui, xi” with breathing and pronunciation. These exercises significantly enhance the function of the liver, heart, spleen, lungs, kidneys, and trifocal organs, respectively, and LQG helps to balance the energy and function of the internal organs [14, 15]. The method includes breathing in through the nose and exhaling with enunciation of one of the six different voices and breathing sound [16].

Recent studies have shown that the six-character formula can effectively improve lung ventilation function in patients with chronic obstructive pulmonary disease (COPD) and improve their daily quality of life. It can enhance the cardiopulmonary function of patients with arrhythmia [15, 17]. Studies have shown that LQG improves symptoms, motor function, and daily quality of life in patients with Parkinson’s disease [18].

Abnormal breathing patterns are caused by abnormal trunk control. However, abnormal trunk control is related to muscle weakness of not only the hemiplegic side trunk but also the nonhemiplegic trunk. The six-character formula improves diaphragm function through one inhalation and six breaths, strengthens the control of the deep core stable muscle group, and increases the control ability of the bilateral trunk. Therefore, the aim of this research is to compare the clinical efficacy of LQG and traditional core stability exercise in the treatment of stroke patients with abnormal trunk posture.

## Methods/design

### Trial design

**Fig. 1:**
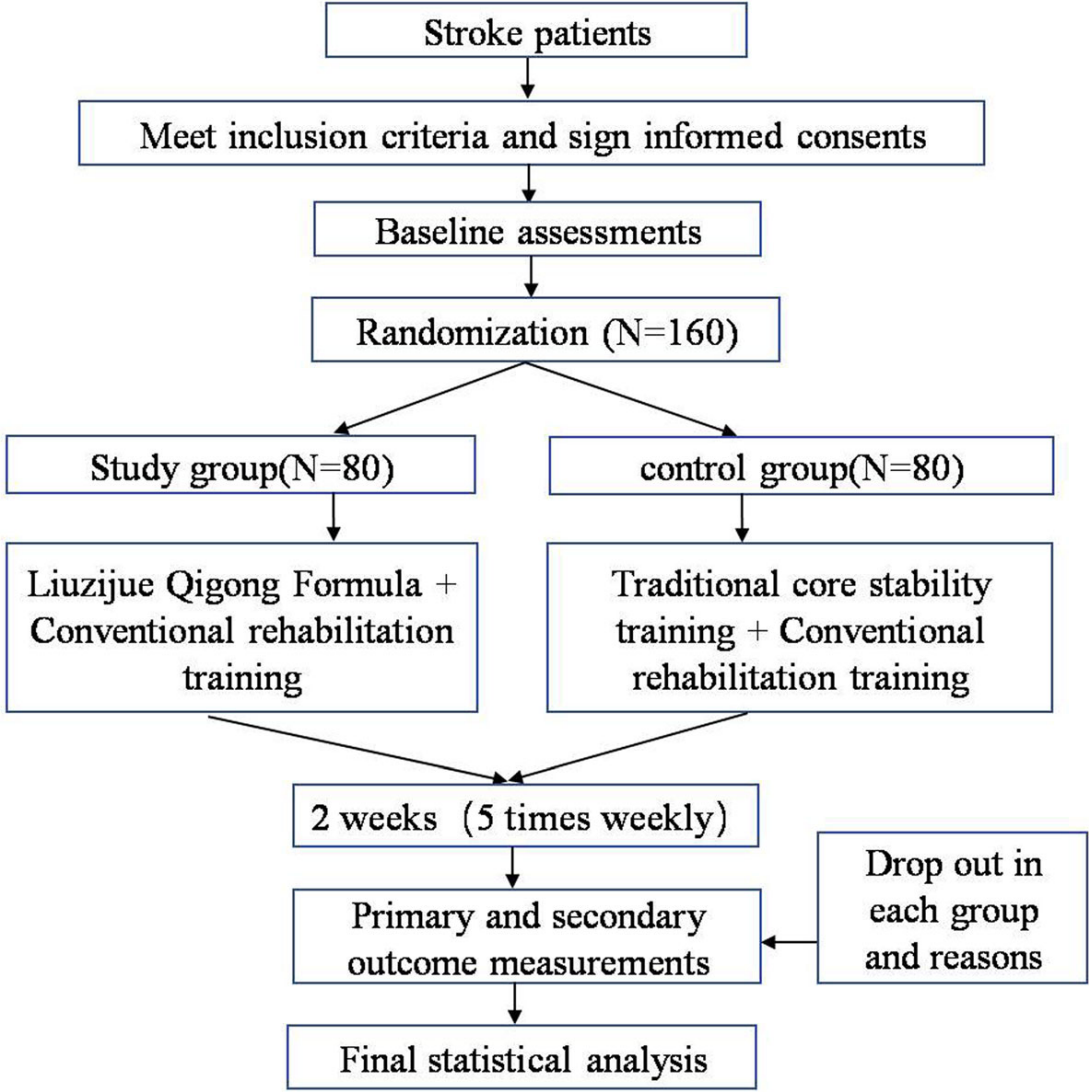
Flowchart of the study

**Fig. 2:**
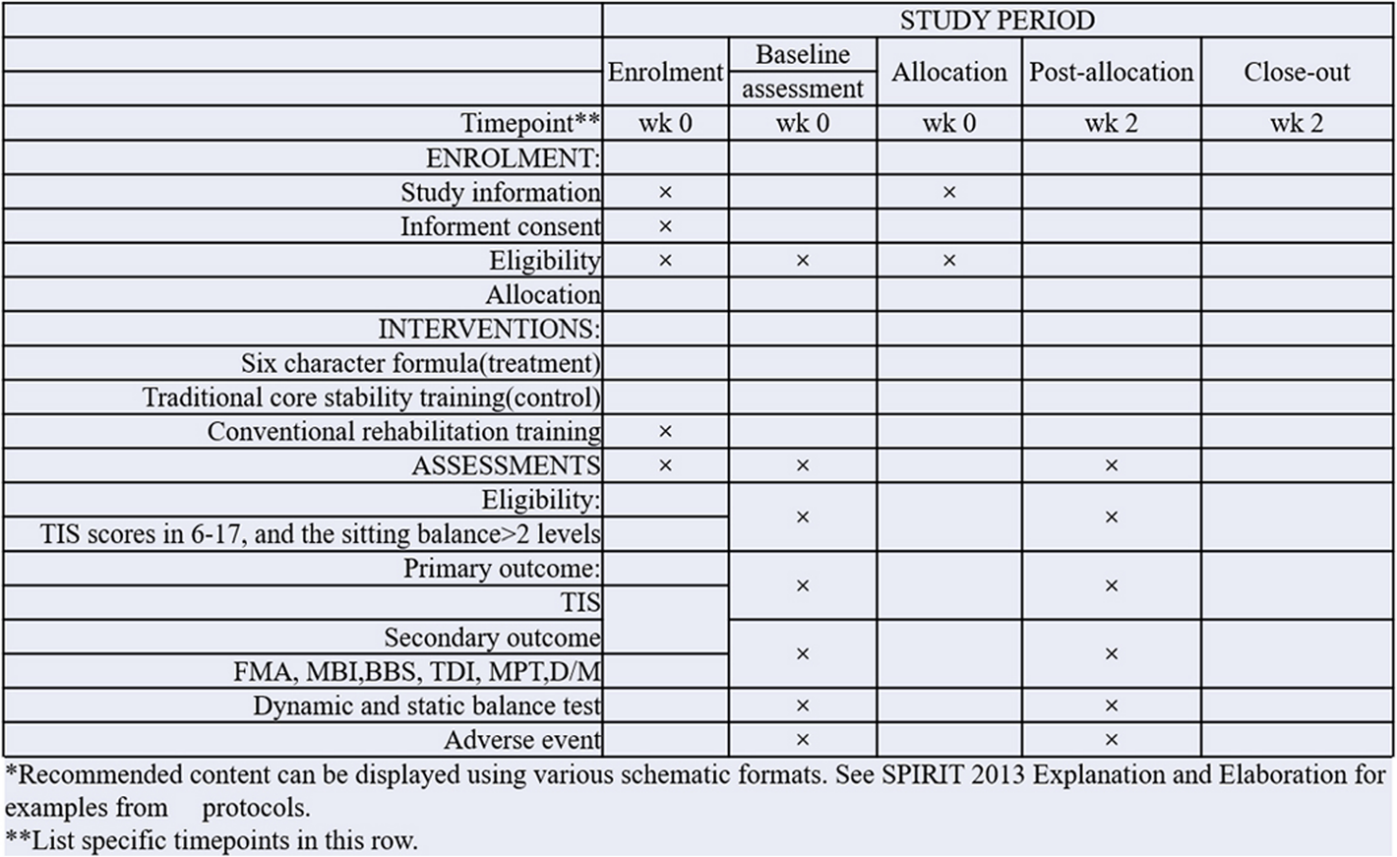
Standard Protocol Items: Recommendations for Interventional Trials (SPIRIT) figure of the LiuZiJue Qigong study protocol

This study will be a single-center randomized controlled trial (RCT) in which 160 stroke patients will be randomly divided into study and control groups. The study will be conducted at Shanghai Xuhui Central Hospital between March 1, 2018, and March 31, 2021. The study will recruit eligible subjects from the inpatient and outpatient departments of rehabilitation medicine of Shanghai Xuhui District Central Hospital in Shanghai. Patients will be allocated in a 1:1 ratio to the study group or the control group. LQG combined with conventional rehabilitation will be completed by patients in the study group, whereas traditional core stability training combined with conventional rehabilitation will be completed by patients in the control group. The Trunk Impairment Scale (TIS) will be used as the main evaluation index. The secondary evaluations index will include the Fugl-Meyer Assessment (FMA), Maximum Phonation Time (MPT), Dynamic and Static Balance Testing (PK-254; TecnoBody, Dalmine, Italy), measurement of the thickness of the diaphragm, measurement of degrees of diaphragm mobility, Modified Barthel Index (MBI), and Berg Balance Scale (BBS). All assessments will be conducted at baseline, at 2 weeks, and at the end of the rehabilitation course. A flowchart of the study protocol is shown in Fig. 1. The research proposal has been designed according to the Standard Protocol Items: Recommendations for Interventional Trials (SPIRIT) 2013 statement recommendations (Fig. 2 and Additional file 1).

### Primary and secondary outcomes

In this study, the TIS will be the main observation index. At the same time, the clinical effect will be divided into three grades to evaluate the curative effect before and after treatment: complete response (CR), partial response (PR), and no response (NR). The results of this observation index will be defined as ranked data.

The secondary outcome measurements will include other relevant evaluation indicators, such as FMA, BBS, ADL, MPT, Dynamic and Static Balance Function, Diaphragm Thickness, and muscle mobility of the diaphragm. The data obtained from these indicators will be measurement data.

### Primary outcome measurement procedures

Experienced physicians will assess the primary measures before and after treatment for 2 weeks. The TIS will be used to measure the main results (Table 1).

**Table 1:** Specific measurement of trunk posture control levels carried out by Trunk Impairment Scale

	Baseline	2 weeks
Trunk posture control measurement		
Evaluation date		
Signature of therapist		

A main investigator will evaluate trunk postural control in the entry and exit stages. Trunk postural control measurement will be conducted according to the Spanish version of TIS 2.0. This scale consists of three subscales. The subscale of the first item is “static sitting balance” (score range 0–7) to assess the ability of patients to maintain sitting balance by landing on their feet and crossing their legs. The patient should cross the leg on the affected side. The second subscale will evaluate dynamic sitting balance (score range 0–10) and assess the lateral flexion of the trunk, starting from the upper and lower trunk. The third subscale will evaluate coordination (score range 0–6) and test the ability to independently rotate the shoulder girdle and pelvic girdle. The total scores will range from 0 to 23, with a higher score denoting better performance [19].

We will evaluate efficacy before and after the intervention According to the following criteria:

CR: TIS total score increased ≥ 7 points after treatment

PR: 3 points ≤ TIS total score increased ≤ 6 points

NR: total TIS score after treatment ≤ 2 points or worse

### Secondary outcome measurement procedures

The secondary outcome measurements will include other relevant evaluation indicators, such as FMA, BBS, ADL, MPT, dynamic and static balance function, diaphragm thickness, and muscle mobility of the diaphragm (Table 2). Experienced physicians will evaluate the secondary outcomes at baseline and 2 weeks after treatment.

**Table 2:** Comprehensive evaluation of trunk posture control disorders after stroke using Fugl-Meyer Assessment, Berg Balance Scale, activities of daily living, maximum phonation time, dynamic and static balance function, diaphragm thickness, and muscle mobility of the diaphragm

	Baseline	2 weeks
Fugl-Meyer Assessment		
Berg Balance Scale		
Barthel Index		
Static balance testing		
1. Displacement difference of the *x* and *y* axes		
2. SD of before and after, left and right directions		
3. Average motion speed before and after, left and right		
4. Motion length		
5. COP (central of pressure) area		
6. Area ratio and length ratio under the test of the motion area and Romberg		
Dynamic balance testing		
1. Trunk stability index (front and rear, left and right, circumference)		
2. Average trajectory error		
3. Test execution time		
4. Average weight force difference (kg)		
Maximum phonation time (s)		
Diaphragm thickness		
Muscle mobility of diaphragm		

*COP* central of pressure

The simplified FMA will be used for the assessment of motor function and is often used in clinical rehabilitation because it is reliable and effective [20, 21]. The scale has a total score of 100, which is divided into motor function evaluation of the upper and lower limbs, among which the upper limb score is 56 and the lower limb score is 44. Each item has a 3-point rating, and a higher score indicates better motor function. The classification of its dysfunction is as follows: < 50 = severe dyskinesia; 50–84 = significant dyspraxia; 85–95 = moderate dyskinesia; 96–99 = mild dyskinesia; and 100 = normal.

The MBI will be used to evaluate the quality of daily life. The scale is divided into ten items and evaluated on the basis of patients’ functional status. The 10 items are eating, bathing, grooming, dressing, controlling bowel movements, controlling urination, toileting, bed and chair transfer, walking on flat ground, and going up and down stairs. The total score is 100 points. The classification of dysfunction is as follows: 100 points = self-care; 61–99 = mild dysfunction; 41–60 = moderate dysfunction; and ≤ 40 = severe dysfunction. The lower the MBI score, the more independent the patients are and the worse their daily living ability [22].

The BBS, which is widely regarded as an effective measure of balance in stroke patients and healthy older adults, will be used to evaluate balance function. The BBS consists of 14 items, each of which is graded on a scale of 0–5. The classification of its dysfunction is as follows: 0–20 points = poor balance function, patient needs to use a wheelchair; 21–40 points = possesses certain balance ability, patient can walk under the assistance; and 41–56 points = good balance function, patient can walk independently. A score < 40 points indicates a risk of falling [23].

MPT will be used to measure the length of time a simple vowel is enunciated after deep breathing. The measurement requirements of MPT are as follows: (1) the longer the pronunciation time, the better; (2) breathing evenly; (3) even breathing loudness; and (4) pitch within the correct frequency range. At the same time, the relatively longer MPT value will be taken as the final evaluation result. For instance, according to the reference standard of Chinese MPT [24]: Males aged between 16 and 40:95% [24.4, 25.2]; Female ages between 16 and 40:95% [16.3, 16.9]. The assessment results must meet the above requirements; namely, the tone and volume of the pronunciation should be kept at a comfortable level [25].

Static stability and dynamic balance testing will be measured by the ProKin proprioception evaluation and training system (PK254P; TecnoBody). Static stability testing will proceed as outlined below [26]:

1. Standard seat position:
  - a. The balance plate is arranged on the platform; the height of the foot platform is adjusted; feet are in contact with the ground; and curtsying subjects sit at 90–100 degrees.
  - b. Adjust the seat of the subject and position the femur large rotor on the balance plate A3–A5 axis.
  - c. Adjustment of the feet to shoulder width of subject
  - d. Chest rise, eyes to the front
  - e. Subject with arms crossed, elbow, shoulder proneness of 45 degrees hung up

The standard standing position will be as follows: (1) symmetrical on the A1–A5 axis and subjects close to each other, and (2) medial border of the feet will be 10 cm apart, and the highest point of the bilateral arch will be located on the A3–A5 axis. The static balance test has six observation indices: (1) displacement difference of the *x* and *y* axes; (2) SD of forward and backward, left and right directions; (3) average motion speed before and after, left and right; (4) motion length; (5) Central of pressure (COP) area; and (6) area ratio and length ratio under the test of the motion area and Romberg test.

Dynamic balance testing will be carried out as follows [27]. The two feet standing apart with inclined plates, entry of general data of patients, selection and evaluation module, and adjustment of the resistance buffer system as recommended by the “8” (a total of 10 files, if the file number is high the more resistance, set to “8”). The tester will be required to wear a chest position sensor, which will be located around the two nipples and connected to the midpoint of the sternum. The dynamic balance test has four observational indices: (1) Trunk Stability Index, front and rear, left and right, circumference; (2) average trajectory error; (3) test execution time; and (4) average weight force difference (in kg).

The thickness of the diaphragm will be measured by ultrasound (Tdi). Subjects will be in the supine position and breathe autonomously. It is worth noting that in order to avoid measurement error, we uniformly selected the right side of patients for measurement. All phrenic ultrasound examinations in this study are performed by the same doctor with formal ultrasound training. Therefore, a linear high-frequency probe will be positioned on the right axillary front and the probe set perpendicular to the chest wall between ribs 8 and 9. If the diaphragm is not visible in this position, the probe may be moved up to the seventh and eighth intercostals. The image of the phrenic junction consists of three layers of connective tissue, namely the bilateral hyperechoic area (pleura, peritoneum) and the intermediate mixed-type echo area (composed of the anechoic diaphragm tissue and its internal hyperechoic fascia). The three layers are parallel during respiration. A moving cursor will be used to measure the thickness of the diaphragm at the end of quiet breathing (functional residual air position; TdiFRC) and the thickness of the diaphragm at the end of maximum inspiration (forced lung capacity; TdiFVC), respectively. The values of three respiratory cycles will be measured and averaged. The change of diaphragm thickness from the end of quiet exhalation to the end of maximum inspiration will be calculated as follows: TF = (TdiFVC − TdiFRC)/TdiFRC [28].

For ultrasound measurement of phrenic muscle mobility, the degree of diaphragm mobility will be measured as the displacement distance of the diaphragm dome FRC and TLC, with subjects lying at a 45-degree angle. A convex transducer (3.5 MHz) will be placed in the medial line of the right axilla at the costal margin of the thorax, and the fixed finger will be positioned toward the skull. For each image of the M-mode record, we will determine the vertical distance between the point corresponding to the starting maximum inspiration and the point corresponding to the maximum diaphragm displacement [29].

### Adverse event collection procedure during the trial

Adverse events and procedures during the trial will be reported, processed, and recorded in a timely manner. This study will complete a 2-week clinical intervention to identify the reasons for adverse events and to ensure that patient safety, health, and rights are maintained. Serious adverse events will be submitted to the principal investigator within 24 h. The clinical trial office and ethics committee of Shanghai Xuhui District Central Hospital will jointly put forward reasonable suggestions.

### Trial setting

The rehabilitation department of Shanghai Xuhui District Central Hospital will carry out this study. Physical therapists will provide comprehensive rehabilitation for all patients, including exercise therapy, occupational therapy, speech therapy, and traditional rehabilitation.

### Inclusion criteria

The following will be criteria for inclusion of subjects in the study:

1. Meet the diagnostic criteria of cerebral infraction or cerebral hemorrhage
2. Meet traditional Chinese medicine (TCM) diagnostic criteria of stroke
3. First onset of stroke, with trunk postural control disorder
4. TIS evaluation score between 6 and 17 and a sitting balance greater than two levels
5. Between 40 and 80 years old
6. Course of disease 2 weeks to 6 months
7. Good physical strength; can withstand 45 min of training
8. At least one side movement function of a normal limb or Brunnstrom stage ≥ 4
9. Stable vital signs
10. Agree to sign informed consent document

### Exclusion criteria

The following will be exclusion criteria for potential subjects in this study:

1. Consciousness disorder, severe cognitive dysfunction, and hemianopia
2. Concomitant dysarthria or aphasia
3. Physical training cannot be tolerated for 45 min
4. Heart, brain, kidney, and other organs with acute disease
5. Serious mental disorders
6. Modified Mini Mental State Examination score ≤ 23

### Study population and recruitment

The study will recruit 160 patients aged 40–80 years who have been diagnosed with cerebral apoplexy in the rehabilitation department of Xuhui District Central Hospital in Shanghai from March 2018 to March 2021. First, before the start of the study, doctors in the inpatient department of rehabilitation medicine should be informed of the criteria of potential study patients who need to be included in the study, as well as the purpose of the study, and then they should be referred to the researchers of the study. Second, the principal investigator will briefly introduce the inclusion and exclusion criteria for this study and provide the researchers with possible inclusion information, as well as the potential risks and advantages. Third, If patient is interested in the study, the primary researchers will further provide more detailed information and answer any questions raised. If the patient agrees to participate in the study, the patient or the patient’s family members will sign the informed consent to guarantee the patient’s privacy. Finally, after the patients and their families have signed the informed consent form, the main researchers will include subjects who meet the requirements according to the TIS.

### Randomization and blinding

#### Randomization

CW generated the allocation sequence; CW and LY will enroll the participants; and YZ will assign participants to the interventions. In this study, a statistician who was not involved in the study used the IBM SPSS Statistics version 20.0 software (IBM Corp., Armonk, NY, USA) to generate a table of random numbers. Patients were randomly divided into two groups at a 1:1 ratio. One group was the study group, and the other was the control group. A table of random numbers will be placed in sequentially labeled opaque envelopes. The distribution order is then kept by a research assistant who is not involved in recruitment, intervention, outcome evaluation, or statistical analysis. After the subjects have met the study criteria, the research assistant will notify the appropriate therapist to intervene and will notify the subject by telephone of the assignment (study group or control group).

#### Blinding

Patients will be randomly assigned through random coding generated by the IBM SPSS Statistics software to ensure that the evaluator is blinded. Only the major researchers will know the order of the random assignments. Outcome assessors will be blinded; that is, the outcome assessors and care providers will be different doctors. The outcome assessors and care providers shall not exchange information in the implementation of the experiments, and the outcome assessors shall not ask the subject for the intervention. In this study, only the assessors will be blinded, so the study does not involve unblinding.

#### Intervention

The LQG (study group) or core stability training (control group) followed by conventional rehabilitation training (30 min each) will be completed in 2 weeks, five times in a quiet 10-m^2^ room with a background noise level ≤ 30 dB. Experienced physiotherapists will be responsible for completing the training and ensuring that the training process is conducted strictly in accordance with the research program. First, the patients should adjust to a sitting and independent standing position. Then, they should breathe smoothly through their nose, complete the upper body movements, and slowly exhale through the mouth. Meanwhile, it is worth noting that this study will not cause any side effects to patients, not negative effects, but in the process of the study, if a patient’s condition worsens, the damage caused by treatment or evaluation shall be borne by national health insurance. All patients will receive comprehensive rehabilitation provided by physiotherapists in the Shanghai Xuhui Central Hospital Department of Rehabilitation Medicine, including exercise therapy, occupational therapy, activities of daily living training, and traditional physical therapy. The intensity and frequency of comprehensive rehabilitation programs received by the two groups of patients will be the same.

#### Control group

Patients in the control group will receive traditional core stability training and conventional rehabilitation therapies. Routine rehabilitation training will include drafting training, passive joint movement, walking between parallel bars, and occupational therapy, among others. At the same time, core stability training will include abdominal training in the supine position, double-bridge movements, single-bridge movements, balance ball half-bridge movements, and forward and backward movement of the sitting pelvis. The patient will be sat on a Bobath ball and use the Bobath handshake to control the flexion, extension, lateral flexion, and rotation of the torso. Before the training begins, the therapist will inform the patient of the brief action instructions. For patients with poor respiratory control, manipulation will be adopted to stimulate their abdominal muscle group contraction, and necessary manipulation should be undertaken according to the different limb functions of the patients. The duration of conventional rehabilitation training will be five times per week, 30 min per session, lasting for 2 weeks. The traditional core stability training will last for 15 min, five times per week, for 2 weeks. All of the control group interventions for traditional core stabilization training will be performed separately by three experienced therapists.

#### Study group

In addition to receiving conventional rehabilitation training, patients in the study group will be required to complete the LQG treatment. Patients allocated to the study group will be engaged in LQG rehabilitation programs in addition to conventional rehabilitation therapies. The sequence will be routine rehabilitation and then core stability training. Each time, the rehabilitation course will be divided into LQG for 15 min and normal rehabilitation training for 30 min. The study group will complete training five times per week for 2 weeks in a quiet room. For traditional core stabilization training, all study group interventions will be performed separately by three experienced therapists.

The key points of LQG oral guidance will consist of pronunciation and breathing. For “xu,” pronunciation will be assisted by the teeth. Space will be left between the teeth and the tongue, with the upper and lower teeth parallel. Air will be drawn from the space between the teeth, as well as between the teeth and the tongue, and the corners of the mouth will be pulled back a little. When exhaling and pronouncing, the “he” will be pronounced with the aid of the tongue. The upper teeth will be tapped with the sides of the tongue and air exhaled between the tongue and the upper jaw. During the exhalation and pronunciation, the “hu” sound will be assisted by the throat. The sides of the tongue will be bent upward and the lips forward, forming a circular opening through which the patient will exhale. During exhalation and pronunciation, the “si” will be pronounced with the assistance of the teeth. There will be a narrow gap between the upper and lower teeth. The patients will lightly touch their lower teeth with the tip of their tongue and exhale air between their teeth. During the exhalation and pronunciation, the “chui” will be pronounced with the assistance of the lips. The tongue and the corner of the mouth will be pulled back, as will the lips into the stretched position, making the back teeth parallel and thus exhaling air from the throat between the sides of the tongue and the stretched lips. In the exhalation process, the “xi” will be pronounced with the assistance of the teeth. The lower teeth will touch the tip of the tongue, and the corners of the mouth will be slightly tilted back, the posterior teeth gently closed, and air exhaled through the space between the posterior teeth [30–32].

Our training is a one-to-one intervention delivered by the therapist. Before training, for the elderly, we will tell each patient that the emphasis will be on breathing and pronunciation. In order that the patient should learn correct pronunciation and breathe accurately, the patient will need to learn gradually to “feel” the key points of strength rather than the absolute power of pronunciation. For example, the patient may complete the training in a sitting or standing position. Stroke patients should learn to use healthy upper limbs to assist upper limb movement. Alternatively, the physiotherapist can stand on the patient’s side to assist in the completion of the movement and maintain the normal position of the chest, spine, and pelvis to provide a stable mechanical structure. Our training is not in the form of group exercises, but in the form of one-to-one therapist intervention.

#### LQG action points

Stroke patients with postural dysplasia often show different degrees of hemiplegia and postural abnormalities, resulting in the loss of their ability to sit or stand. It is impossible for these patients to complete the LiuZiJue key independently and precisely without assistance. Therefore, the therapist will properly guide the completion of the movement and adjust the training position according to the patient’s functional state. As the improvement of patient’s balance ability increases, the training position will gradually transition from the sitting position to an independent standing position. As the patient’s limb function improves, the therapist will gradually reduce the amount of assistance. Both active and auxiliary limb movements are based on bilateral limb opposition. The most important goal is to complete an accurate respiratory guidance program.

#### Sample size calculation

We will use the TIS as the main therapeutic index to evaluate trunk posture control. The preliminary experimental findings showed that the effective rate was 80% in the study group and 50% in the control group. The effective rates of the study group and the control group were set as 80% and 50%, respectively. On this basis, sample estimation was conducted using G*Power 3.1 software, and the rank-sum test was adopted to set the rates of 80% in the treatment group and 50% in the control group for bilateral testing, and we set α = 0.05 and power = 0.97 (the setting of this value is based on the statistical power after the end of the experiment; power = 0.97 after the end of 40 cases). The distribution ratio was matched by 1:1, and the final sample size was set as 142, with 71 subjects in each of the two groups, taking into account the possibility of 12% attrition rate during the study. Therefore, the total number of samples was finally determined to be 160 cases, namely 80 cases in each group.

### Statistical analysis

In this study, the main indicators were determined according to the change levels of TIS before and after treatment, which were divided into three levels of CR, PR, and NR and expressed by rate or percentage. The chi-square test will be used to compare differences in efficacy between groups. The overall curative effect evaluation and the curative effect evaluation method will be employed. The calculation formula is as follows: Effective = (excellent + effective)/total cases × 100%. Secondary indicators (e.g., MPT, MBI, BBS, FMA, diaphragm thickness and mobility, static and dynamic balance ability) are all continuous data. A paired sample *t* test is often used for intragroup comparison, whereas an independent sample *t* test is often used for intergroup comparison of the difference between the two groups after treatment, and a two-sided test will be used with the significance level set at *P* ≤ 0.05. To eliminate the influence of rehabilitation intervention time, stroke type, age, and gender on the efficacy and relative size of intervention methods will be considered and the statistical method of stepwise logistic regression model adopted.

The intention-to-treat (ITT) group is divided into final analysis set (FAS) and per protocol (PP), where FAS refers to the data set obtained after minimal and fair removal of data from all randomized subjects. PP is sometimes referred to as “valid case” and “valid sample” and is a subset of the total analysis set. The subjects had sufficient adherence to the protocol to be able to estimate the effect of the treatment.

The PP population will include random patients in the ITT group but will exclude patients who do not meet the inclusion or exclusion criteria and randomly assigned patients who do not receive the actual treatment [33].

If the statistical results are the same, ITT and PP are reliable indicators. ITT results will be used if statistical evaluations are different between the two methods. As the final observation result (last observation carried forward), the missing data will be processed. The last observation value of the endpoint will be regarded as the follow-up evaluation point of missing data, and the last observation reaction will be regarded as the endpoint of the study.

#### Monitoring

To ensure the quality of this RCT, this study will be completed by Shanghai Xuhui Central Hospital. We will upload data through the Chinese Clinical Trial Register in a timely manner so that the project management team can identify problems, review collected data, and control errors. The drug testing center of Shanghai Xuhui Central Hospital will have the opportunity to get the results of the midterm trial and make a decision on it. A qualified clinical trial specialist will be invited to monitor the RCT.

#### Trial quality control

The main researchers, CW and YZ, are responsible for making protocol decisions, whereas LY is responsible for coordination (e.g., collating/collecting data, analyses). JY will be responsible for quality control; YNZ and RWW will be responsible for data development and database management; and YZ will be responsible for setting up a quality control committee.

#### Researcher training

All researchers will receive good clinical practice training and have clinical expertise, qualifications, and appropriate abilities for the study. Prior to the beginning of the project, all subjects enrolled will receive uniform training. Through program training in clinical research, the clinical research purpose will be fully understood by all researchers, including the plan, indicators, and case report form (CRF) documents. Each researcher will be issued a “researcher’s manual” as a reference guide.

#### Data management

This study will be conducted in strict accordance with the phase plan of the trial. After the baseline and 2-week interventions, all data will be recorded in the CRF, and then the data in the CRF will be uniformly input into Excel spreadsheets (Microsoft Corp., Redmond, WA, USA) by CW and YNZ. We will create a separate folder for storing the data. At the same time, the data will also be transferred to the network disk for backup, so as not to lose data. We also set up a data management team for two people. When there are errors, one person is responsible for finding and correcting the errors, and the other person is responsible for checking the data again.

#### Ethics

This study has been approved by the ethics committee of the Shanghai Xuhui District Central Hospital (no. 2017040) and strictly follows the principles of the Helsinki declaration and statement. The trial is registered with the Chinese Clinical Trial Register (ChiCTR1800014864). All participants will fully understand the contents of this agreement and sign the informed consent document. To determine whether the members of the committee find it necessary to change the research plan, we will submit a detailed written application to the ethics committee.

## Discussion

Abnormal breathing patterns and trunk posture affect each other in stroke patients [34]. A study has confirmed that breathing training can significantly increase muscle strength and trunk coordination in stroke patients with hemiplegia and also improve respiratory function [35, 36]. It has been reported that respiratory muscle strength training or complex breathing exercises can effectively improve the stability of the trunk posture of stroke patients [37], and it has been proven that resistance training of the thoracic cage can significantly improve respiratory function and the trunk control ability of stroke patients [38]. Nelson proposed that breathing is the foundation of core stability, and core stability is the foundation of movement [39]. Research has shown that abdominal muscle thickness decreases in patients with chronic stroke and that respiratory muscle function is positively correlated with trunk function and balance [40]. Thus, respiratory muscle training should be regarded as part of trunk control training for stroke patients.

LQG is a traditional Chinese breathing training method with breathing and breathing training at its core. Through six words, different mouth parts, lips, teeth, throat, and tongue force differences can affect different visceral flows [13]. LQG requires the maintenance of an upright posture, relaxation of the neck, and the maintenance of normal positions of the thoracic spine and pelvis, which may provide a stable core mechanical environment for stroke patients to exercise correct breathing patterns [41]. Combined with bilateral body and trunk movements, noninvasive opening of the thoracic cavity and increasing the volume of the thorax may be beneficial for the movement of the diaphragm [15]. Combined with one inhalation and six exhalations, it guides stable and continuous air flow and controls internal abdominal pressure, which may “massage” the chest and abdominal viscera, gently activate the weakened core muscle groups, inhibit the overstrained muscle groups, and promote the coordination of trunk core muscles.

We hypothesize that stroke patients with hemiplegia will improve their abnormal trunk posture control through LQG exercises, increase their respiratory control ability, and improve their core stability. We hope that stroke patients can gradually learn to transition from single thoracic breathing to abdominal breathing by practicing the combination of LQG motor guidance and mouth type breathing pronunciation. Only this breathing training method can maximize the coordination of breathing and trunk posture, which is readily accepted by patients. Therefore, the purpose of this study is to compare the effects of LQG with traditional core stability training on trunk posture control after stroke.

The TIS is an evaluation method to test the effects of improvement of main trunk functions through examination of static balance, dynamic balance, and dynamic coordination, which is helpful for neurological diagnosis and treatment guidance [42]. Patients with trunk posture control disorder after stroke will be selected as study subjects through the TIS and other examinations, including BBS, FMA, ADL, MPT, and dynamic and static balance function. A comprehensive evaluation of the effectiveness of the training therapy will be achieved by measuring the thickness of the diaphragm and the movement of the diaphragm muscles.

To sum up, the proposed study will focus on patients with abnormal trunk posture control and intends to introduce LQG into the rehabilitation of abnormal trunk posture control after stroke by ensuring that breathing, speech, and movement guidance are trained synchronously.

### Limitations of the study

The limitations of the proposed study are as follows: (1) Due to the limited medical resources, patients are hospitalized only for a short period of time, with the intervention period of the study being only 2 weeks; (2) no follow-up investigation will be conducted after the intervention; (3) single- or double-blind observations will not be achieved; and (4) surface myoelectricity will not be used to test the activity of the trunk muscle group, and abdominal pressure test tools will not be used to reflect changes in abdominal pressure.

### Trial status

The version number of this trial scheme is the first version, dated 25 December 2017. Updated here for date 1, march 2018. Patient recruitment began on 1 March 2018 and is expected to continue for 3 years; patient recruitment will end on 1 March 2021.

## Supplementary information

**Additional file 1.** SPIRIT 2013 checklist.

## Data Availability

The datasets supporting the conclusions of this article are included in the published article.

## Abbreviations

BBS: Berg Balance Scale
COP: Central of Pressure
COPD: Chronic obstructive pulmonary disease
CR: Complete response
CRF: Case report form
FAS: Final Analysis Set
FMA: Fugl-Meyer Assessment
ITT: Intention to treat
MBI: Modified Barthel Index
MPT: Maximum phonation time
NR: No response
PP: Per protocol
PR: Partial response
RCT: Randomized controlled trial
Tdi: Ultrasound measurement of diaphragm thickness
TIS: Trunk Impairment Scale
